# Ouabain Modulates Zymosan-Induced Peritonitis in Mice

**DOI:** 10.1155/2015/265798

**Published:** 2015-05-11

**Authors:** Jacqueline Alves Leite, Anne Kaliery De Abreu Alves, José Guilherme Marques Galvão, Mariana Pires Teixeira, Luiz Henrique Agra Cavalcante-Silva, Cristoforo Scavone, Alexandre Morrot, Vivian Mary Rumjanek, Sandra Rodrigues-Mascarenhas

**Affiliations:** ^1^Instituto de Ciências Biomédicas, Departamento de Farmacologia, Laboratório de Neurofarmacologia Molecular, Universidade de São Paulo, 05508-900 São Paulo, SP, Brazil; ^2^Centro de Biotecnologia, Laboratório de Imunofarmacologia, Programa de Pós-Graduação em Fisiologia Multicêntrico, Universidade Federal da Paraíba, 58059-900 João Pessoa, PB, Brazil; ^3^Instituto de Bioquímica Médica Leopoldo de Meis, Laboratório de Imunologia Tumoral, Universidade Federal do Rio de Janeiro, 21941-902 Rio de Janeiro, RJ, Brazil; ^4^Laboratório de Biologia do Sistema Imune, Instituto de Microbiologia, Universidade Federal do Rio de Janeiro, 21941-902 Rio de Janeiro, RJ, Brazil; ^5^Centro de Ciências da Saúde, Programa de Pós-Graduação em Produtos Naturais e Sintéticos Bioativos, Universidade Federal da Paraíba, 58059-900 João Pessoa, PB, Brazil

## Abstract

Ouabain, a potent inhibitor of the Na^+^, K^+^-ATPase, was identified as an endogenous substance. Recently, ouabain was shown to affect various immunological processes. We have previously demonstrated the ability of ouabain to modulate inflammation, but little is known about the mechanisms involved. Thus, the aim of the present work is to evaluate the immune modulatory role of ouabain on zymosan-induced peritonitis in mice. Our results show that ouabain decreased plasma exudation (33%). After induction of inflammation, OUA treatment led to a 46% reduction in the total number of cells, as a reflex of a decrease of polymorphonuclear leukocytes, which does not appear to be due to cell death. Furthermore, OUA decreased TNF-*α* (57%) and IL-1*β* (58%) levels, without interfering with IL-6 and IL-10. Also, *in vitro* experiments show that ouabain did not affect endocytic capacity. Moreover, electrophoretic mobility shift assay (EMSA) shows that zymosan treatment increased (85%) NF-*κ*B binding activity and that ouabain reduced (30%) NF-*κ*B binding activity induced by zymosan. Therefore, our data suggest that ouabain modulated acute inflammatory response, reducing the number of cells and cytokines levels in the peritoneal cavity, as well as NF*κ*B activation, suggesting a new mode of action of this substance.

## 1. Introduction

Ouabain, a steroid capable of inhibiting Na^+^K^+^-ATPase, is an adrenal and hypothalamus-derived hormone, present in mammalian plasma and tissue, which appears to be produced in response to volume expansion, angiotensin II, and/or adrenocorticotropic hormone stimulation [1–4]. It has also been suggested that endogenous ouabain levels are modulated by stress conditions [5, 6]. Moreover, elevated levels of ouabain were found in hypertensive patients and normotensive individuals after physical exercise [5, 7].

Ouabain is able to modulate several immunological functions, inhibiting* in vitro* proliferation of lymphocytes induced by different mitogens [8–11], increasing the expression of the molecule CD69 on the thymocyte surface [12], and decreasing the levels of phosphorylated MAPK p38 and nuclear factor of activated T cells (NFATc1) [13]. The combination of ouabain plus corticoids produced a synergic effect inducing thymic atrophy* in vivo* [14]. Additionally, ouabain was shown to modulate function and cytokine production of monocytes* in vitro* [15] and dendritic cells [16]. Ouabain negatively modulated acute peritoneal inflammatory response induced by* Leishmania amazonensis* infection in Swiss mice [17]. It was also reported that ouabain presented anti-inflammatory and antinociceptive effects [18], but the mechanisms involved were still unclear. Nonetheless, some ouabain effects are controversial. It has also been described, using different models, that ouabain is capable of increasing the activation of NF-*κ*B, VCAM-1, and iNOS expression, NO, and inflammatory cytokines levels [19–22].

During inflammation, a complex process of intracellular signal transduction and transcription events, driven by multiple proinflammatory mediators and cytokines, is activated. Acute inflammation is characterized by exudation of protein-rich fluid, edema, vasodilatation, and cell migration, into the site of injury [23]. Intraperitoneal injection of zymosan, a polysaccharide component of cell wall from* Saccharomyces cerevisiae,* represents a self-resolving model of acute inflammation and has been widely used for the quantification of particular cell types and inflammation-related soluble factors [24–26]. When administered in mouse peritoneal cavity, zymosan induces an increased vascular extravasation, which is one of the primary signs of inflammation. This is a key step in inflammatory exudate formation and is followed by a time-dependent recruitment of migratory cells, specially neutrophils and monocytes/macrophages [26–32]. Therefore, to better understand the anti-inflammatory effect of ouabain, we aimed to assess its action in vascular leakage, leukocyte migration, as well as apoptosis of leukocytes, cytokine production, and endocytosis.

## 2. Material and Methods

### 2.1. Animals

Female Swiss albino mice (2 months old) were obtained from Thomas George animal house of Centro de Biotecnologia (CBiotec, UFPB, João Pessoa, Brazil). Animals were kept under standard laboratory conditions on a constant 12 h light/dark cycle with temperature (21 ± 1°C). Food and water were given* ad libitum*. All procedures adopted in this study were approved by Institutional Ethics Committee of Centro de Biotecnologia (Protocol: 0504/11).

### 2.2. *In Vivo* Treatment with Ouabain

In all* in vivo* experiments, 0.56 mg/kg ouabain (Sigma Aldrich) [14, 17, 18] or phosphate buffered saline (PBS) was given intraperitoneally (i.p.) for three consecutive days.

### 2.3. Assessment of Vascular Permeability

Mice were randomly divided into four groups (*n* = 6) and each group of mice was administrated intraperitoneally (i.p.) once a day for 3 consecutive days with either vehicle (PBS) or ouabain (0.56 mg/kg). One hour after the last injection on day 3, mice were injected i.v. into the caudal vein with Evans blue dye suspended in saline at 10 mg/mL (0.3 mL/mouse). The dye injection was immediately followed by an i.p. injection of zymosan. The animals were euthanized after 30 min and the peritoneal cavity was lavaged with 1.5 mL of saline. Lavage fluid was then centrifuged for 10 min at 350 g. The absorbance of cell-free lavage fluid was measured at 650 nm [32].

### 2.4. Induction of Peritonitis

Peritoneal inflammation was induced as previously described [26, 32]. Mice were randomly divided into four groups (*n* = 8), and each group received i.p. the same volume (200 *μ*L) of vehicle (PBS) or ouabain (0.56 mg/kg) once a day for 3 consecutive days. Zymosan A was freshly prepared (2 mg/mL) in sterile 0.9% w/v saline and 0.5 mL was injected i.p. one hour after the last injection on day 3. At either four or twenty-four hours after stimulus, animals were euthanized by cervical dislocation. The peritoneal cavity was then lavaged with 1 mL of cold PBS. Exudates were pooled and the number of leukocytes present was determined by optical microscopy in Hemocytometer, using Turk's solution (0.01% crystal violet in 3% acetic acid). Finally, the exudates were centrifuged and an aliquot of the supernatant was collected and stored at −20°C for cytokine analysis.

### 2.5. Leukocytes Subsets

Analysis of the cell populations in the peritoneal cavity was performed by flow cytometry, based on size and granularity parameters, as well as on Gr-1 (Ly-6G and Ly-6C) expression and Mac-3 expression, to identify neutrophils and macrophages, respectively. Monocytes expressed both Gr-1 and Mac-3 (Gr1^+^Mac3^+^). Leukocytes collected from peritoneal lavage were stained with the antibodies kits (eBioScience) according to the manufacturer's protocol. Briefly, 1 × 10^6^ cells/mL were incubated with a saturating amount of anti-Gr-1 PE-conjugated and anti-Mac-3 FITC-conjugated (eBioScience) for 30 min at 4°C. Cells were then washed with cold PBS and resuspended in the same solution. Analyses of Gr-1 and Mac-3 expression were performed using FACSCalibur flow cytometer. In these experiments, data were acquired in a mode of 10,000 events. FITC was measured in FL-1 and PE was measured in FL-2 channels. Data were analyzed by WinMID software.

### 2.6. Measurement of Cytokines Levels by an Enzyme-Linked Immunosorbent Assay (ELISA)

TNF-*α*, IL-1*β*, IL-6, and IL-10 levels in the peritoneal fluid were evaluated by mouse specific sandwich ELISA, according to the manufacturer's instructions (eBioScience). Optical density was read using a microplate spectrophotometer (microplate reader VersaMax, tunable, BN 2529, Molecular Devices).

### 2.7. Apoptosis Assessment by Flow Cytometry

Apoptotic leukocytes were identified quantitatively by Annexin V-PE Apoptosis Detection Kit (BD Bioscience) that enables cell staining with Annexin V and propidium iodide (PI). Annexin V binds to phosphatidylserine exposed on the outer leaflet of the plasma membrane of apoptotic dying cells while PI is a vital dye that enters any necrotic cell. Leukocytes collected from peritoneum were stained with this kit, according to the manufacturer's protocol. Briefly, cells were washed twice with cold PBS and resuspended in binding buffer (10 mM Hepes, 140 mM NaCl, and 25 mM CaCl_2_). Then, 2.5 *μ*L of Annexin V-PE and 10 *μ*L of PI (50 g/mL) were added to cell suspensions (5 × 10^5^ cells/100 *μ*L binding buffer) and incubated for 15 min at room temperature in the dark. Fluorescence measurement was performed using a flow cytometer (BD FACSCalibur). Annexin V-PE was measured in FL-2 and PI in FL-1 channels. Results were analyzed by WinMID software.

### 2.8. Analysis of Macrophage Viability

Peritoneal exudate was elicited in mice with an i.p. injection of 3 mg/mL of thioglycolate (Sigma Aldrich). Four days after the i.p. thioglycolate injection, animals were euthanized by cervical displacement and the peritoneal cavity was washed with 8 mL of complete RPMI-1640 medium (Gibco) (streptomycin: 10 mg/mL, penicillin: 6 mg/mL, and kanamycin: 2 mg/mL), supplemented with 10% fetal bovine serum (FBS) (Gibco). Cell suspension obtained from peritoneal lavage was centrifuged at 350 g for 15 minutes (4°C). Supernatant was discarded and the pellet was resuspended in 1 mL of complete RPMI medium. Viable cells were counted with a Neubauer chamber (Hemocytometer L. Optik ATC-111020) using Trypan blue solution (Merck). Macrophages were then enriched by adherence to plastic. For that, viable peritoneal cells were seeded in 96-well plates at a concentration of 4 × 10^5^ cells/well in a final volume of 200 *μ*L and incubated for 2 h with FBS-free culture medium in an atmosphere of 5% CO_2_ at 37°C. Then, nonadherent cells were removed by wash with PBS. Remaining cells were further incubated for 24 h with complete RPMI medium, in the presence and absence of different concentrations of ouabain (1, 10, and 100 nM). Cell viability was estimated by MTT (3-[4,5-dimethylthiazol-2-yl]-2,5-diphenyltetrazolium bromide) assay. For that, 200 *μ*L of RPMI medium containing 20 *μ*L of MTT solution (5 mg/mL of MTT in PBS) was added to each well. After 4 h of incubation, MTT-containing medium was removed and the precipitate was solubilized in DMSO solution (200 *μ*L). The optical density was read at 570 nm using a microplate spectrophotometer (SpectraMax; Molecular Devices, Sunnyvale, CA).

### 2.9. Quantification of Fluorescein Isothiocyanate- (FITC-) Dextran Endocytosis by Flow Cytometry Analysis

Resident peritoneal cells were placed in sterile 24-well plates, at the concentration of 2 × 10^6^ cells/mL (final volume = 0.5 mL), and incubated for 2 h with RPMI-1640 medium containing 10% FCS in an atmosphere of 5% CO_2_ and 37°C. Following the incubation period, cells were washed twice with PBS for the removal of nonadherent cells. Remaining cells were further incubated in a final volume (500 *μ*L) of RPMI-1640 medium containing 10% FCS with 0.5 mg/mL of 40 kDa FITC-dextran (Sigma Aldrich), in the presence or absence of different concentrations of ouabain (1, 10, and 100 nM), for 1 h at 37°C or 4°C (control endocytic activity), or in RPMI-1640 medium containing 10% FCS, in the presence or absence of different concentrations of ouabain, for 24 h at 37°C followed by the addition of RPMI-1640 medium containing 10% FCS with FITC-dextran (0.5 mg/mL) for 1 h at 37°C or 4°C. Cells were then washed with ice-cold PBS and fluorescence measurement was performed using a BD FACSCalibur flow cytometer. Fluorescence signs corresponding to dextran-FITC uptake were analyzed by Summit v4.3 software (Dako, USA) using a macrophage/neutrophil gate, which was determined according to cell size and complexity parameters. In a manner somewhat similar to the previous article from Teixeira and Rumjanek [15], results are presented both as percentage of cells that endocytosed dextran and as MFI (median fluorescence intensity) a measurement that, in the present work, is representative of the amount of fluorescence per cell.

### 2.10. Transmigration Assay

Two different groups of cells were tested for their migratory capacity* in vitro*. The control group consisted of resident peritoneal cells obtained from animals injected, once a day, in the three previous days with PBS and the experimental pool obtained from animals injected with ouabain (0.56 mg/kg) once a day for 3 consecutive days. Chemotaxis was measured by migration through a polycarbonate filter of 5 *μ*m pore size in 24-well transwell chambers (Corning Costar, Cambridge, MA). DMEM containing 0.5% FCS (500 *μ*L) plus lipopolysaccharide (100 ng/mL), or medium alone as control for spontaneous migration, was added to the lower chambers. 10^6^ cells (100 *μ*L) were added to the upper chambers and were incubated at 37°C in a 5% CO_2_ humidified atmosphere. After 4 h, migration was defined by counting the cells that migrated to the lower chambers by flow cytometry (BD FACSCalibur flow cytometer).

### 2.11. Nuclear Extracts

Nuclear extracts of peritoneal cells were prepared based on an adapted version of Rong and Baudry [33]. Briefly, cells were lysed in cold phosphate buffered saline (PBS) [supplemented with 2 *μ*g/mL leupeptin, 2 *μ*g/mL antipain, and 0.5 mM PMSF (all obtained from Sigma Aldrich)] and centrifuged at 4°C for 2 min at 12,000 g. Pellets were resuspended in lysis buffer [10 mM HEPES pH 7.9, 1.5 mM MgCl_2_, 10 mM KCl, 0.1 mM EDTA, 0.5 mM PMSF, 2 *μ*g/mL leupeptin, 2 *μ*g/mL antipain, 3 mM sodium orthovanadate, 30 mM sodium fluoride, and 20 mM sodium pyrophosphate (all obtained from Sigma Aldrich)] and incubated on ice for 15 min. After that, NP-40 (Sigma Aldrich) (final concentration of 0.5%) was added, and samples were vigorously mixed and centrifuged for 30 s at 12,000 g. Pellets were then resuspended in extraction buffer [20 mM HEPES, pH 7.9, 25% glycerol, 1.5 mM MgCl_2_, 300 mM NaCl, 0.25 mM EDTA, 0.5 mM PMSF, 2 *μ*g/mL leupeptin, and 2 *μ*g/mL antipain (all obtained from Sigma Aldrich)], incubated for 20 min on ice, and centrifuged for 20 min at 13,000 g at 4°C. The resulting supernatants containing nuclear proteins were stored at −80°C. Protein concentration was determined using the Bio-Rad (Richmond, CA, USA) colorimetric assay [34].

### 2.12. Electrophoretic Mobility Shift Assay

Electrophoretic mobility shift assay (EMSA) for NF-*κ*B was performed using gel shift assay kit from Promega (Madison, WI, USA) [33]. NF*κ*B double-stranded consensus oligonucleotide (5′-AGTTGAGGGGACTTTCCCAGGC-3′) was end-labeled with *γ*-32P-ATP using T4 polynucleotide kinase. Unincorporated nucleotides were removed by passing the reaction mixture through a Sephadex G-25 spin column (Amersham-Pharmacia, Uppsala, Sweden). Purified 32P-labeled probe (25,000 cpm) was incubated in 20 *μ*L with 5 *μ*g of nuclear extracts in a binding reaction mixture containing 50 mM NaCl, 0.2 mM EDTA, 0.5 mM DTT, 4% glycerol, 10 mM Tris–HCl (pH 7.5), and 0.05 *μ*g of poly (dI–dC) for 30 min at room temperature. DNA-protein complexes were separated by electrophoresis using a 6% nondenaturing acrylamide: bisacrylamide (37.5 : 1) gel in 0.5 X Tris-borate/EDTA (TBE) for 2 h at 150 V. Gels were vacuum dried and analyzed by autoradiography. Autoradiographs were visualized with a photo documentation system DP-001-FDC (Vilber Lourmat, Marne la Vallée, France) and quantified in NIH ImageJ software (Bethesda, MD, USA). Several exposure times were analyzed to ensure the linearity of the band intensities.

### 2.13. Statistical Analysis

All data were expressed as mean ± SEM and analyzed by Graphpad Prism 5.0 software using one-way analysis of variance (ANOVA) followed by Tukey's test. Results were considered statistically significant when *P* < 0.05.

## 3. Results

### 3.1. Ouabain Decreased Zymosan-Induced Plasma Extravasation

**Figure 1:**
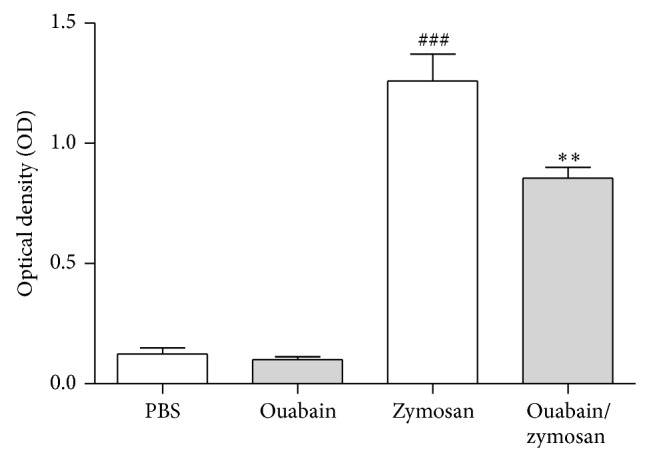
Ouabain decreases plasma extravasation induced by zymosan. Swiss mice (*n* = 6) were pretreated with 0.56 mg/kg of ouabain (i.p.) or PBS for 3 consecutive days. One hour after the last dose of treatment, animals were stimulated with zymosan (i.p.). The results were expressed as absorbance for the Evans blue dye present in the peritoneal exudate. Results were expressed as mean ± SEM and analyzed by Graphpad Prism using ANOVA with Tukey's posttest, where all groups were compared. ^###^ *P* < 0.001 versus PBS group and ^∗∗^ *P* < 0.01 versus zymosan group.

Figure 1 shows that zymosan administration led to an increase (90%) on early vascular permeability, measured at 30 min, when compared to PBS control group. Pretreatment with ouabain (0.56 mg/kg i.p.) for three consecutive days reduced (33%) plasma extravasation induced by zymosan. As expected, pretreatment for three consecutive days with PBS without ouabain had no effect on vascular permeability.

### 3.2. Ouabain Decreased Peritonitis Induced by Zymosan

**Figure 2:**
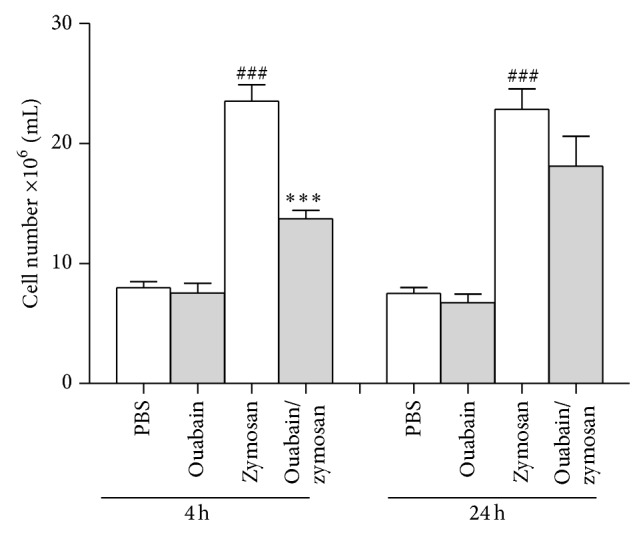
Total cell number in the peritoneal cavity 4 and 24 h after injection with zymosan. Swiss mice (*n* = 8) were treated with 0.56 mg/kg of ouabain (i.p.) or PBS for 3 consecutive days. One hour after the last dose of treatment, animals were stimulated with zymosan (i.p.). Four and 24 hours after challenge with zymosan, peritoneal exudate was collected and total leukocyte counts were performed. Results were expressed as mean ± SEM and analyzed by Graphpad Prism using ANOVA with Tukey's posttest, where all groups were compared. ^###^ *P* < 0.001 versus PBS group and ^∗∗∗^ *P* < 0.01 versus zymosan group.

Peritonitis was measured 4 and 24 h after zymosan treatment. As shown in Figure 2, administration of ouabain had no effect on the number of resident peritoneal cells. However, zymosan treatment led to an increase in total cell number in the peritoneal cavity at 4 and 24 h (66% and 67%, resp.). Ouabain was able to inhibit zymosan-induced leukocyte number in the peritoneal cavity at 4 h (46%), but not at 24 h, when compared to the zymosan group.

### 3.3. Leukocytes Subsets in Peritoneum

**Figure 3:**
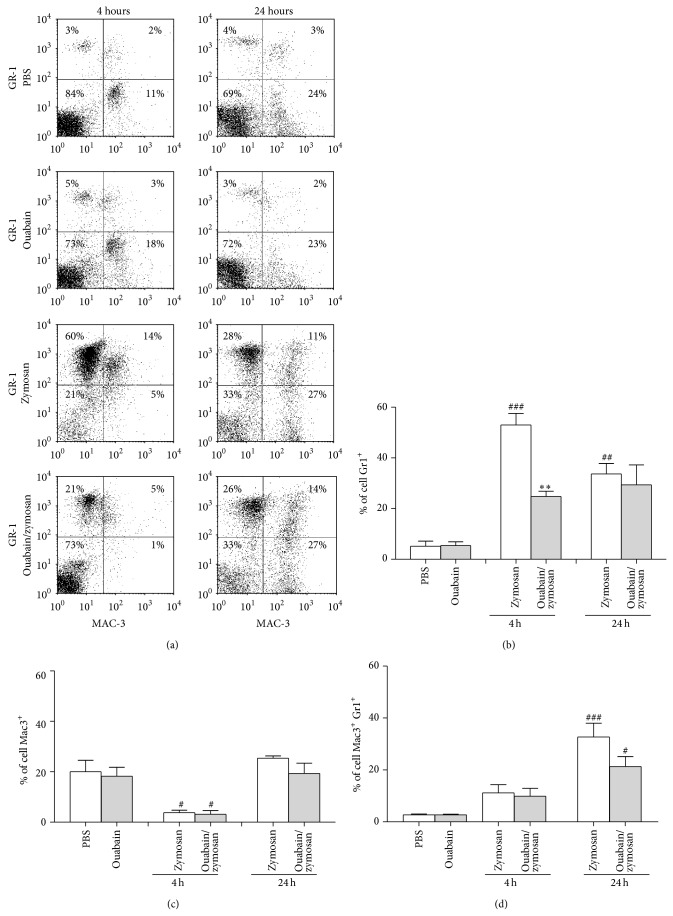
Effect of ouabain on peritoneal cavity leukocyte subsets. Cells were obtained from peritoneal lavage, 4 and 24 h after the injection of the stimuli i.p. Neutrophils, monocytes, and macrophages were distinguished by flow cytometry using anti-Gr-1 and anti-Mac-3 antibodies. (a) Representative experiment showing the patterns of Gr-1^+^ and Mac-3 expression, as well as the leukocyte populations defined by these molecules. Values of the percentage of neutrophils (Gr-1^+^), macrophages (Mac-3^+^), and monocytes (Gr-1^+^ and Mac-3^+^) were also added to the* dot plots*. ((b), (c), and (d)) Data were expressed as mean ± SEM and analyzed by Graphpad Prism using ANOVA with Tukey's posttest, where all groups were compared. The results were obtained from 6 animals per group. ^###^ *P* < 0.001 and ^#^ *P* < 0.05 versus PBS group; ^∗∗^ *P* < 0.01 versus zymosan group.

Once we determined that ouabain reduces peritoneal exudate leukocyte number, flow cytometry was used to identify which cell subpopulations were affected at 4 and 24 h after zymosan treatment. As expected, no significant difference in cell subpopulations was observed between PBS and ouabain groups (Figures 3(a), 3(b), 3(c), and 3(d)). However, four hours after zymosan i.p. injection, there was a significant increase (90%) in neutrophils (Gr1^+^) at the site. Also, ouabain pretreatment was able to reduce neutrophil cell population (53%) when compared to zymosan group (Figure 3(b)). Monocyte (Gr1^+^Mac3^+^) and macrophage (Mac3^+^) cell numbers were only enhanced significantly 24 h after zymosan injection; at this time-point, ouabain pretreatment was not able to inhibit monocyte or macrophages cell number (Figures 3(c) and 3(d)).

### 3.4. Ouabain Modulates Cytokines Induced by Zymosan

**Figure 4:**
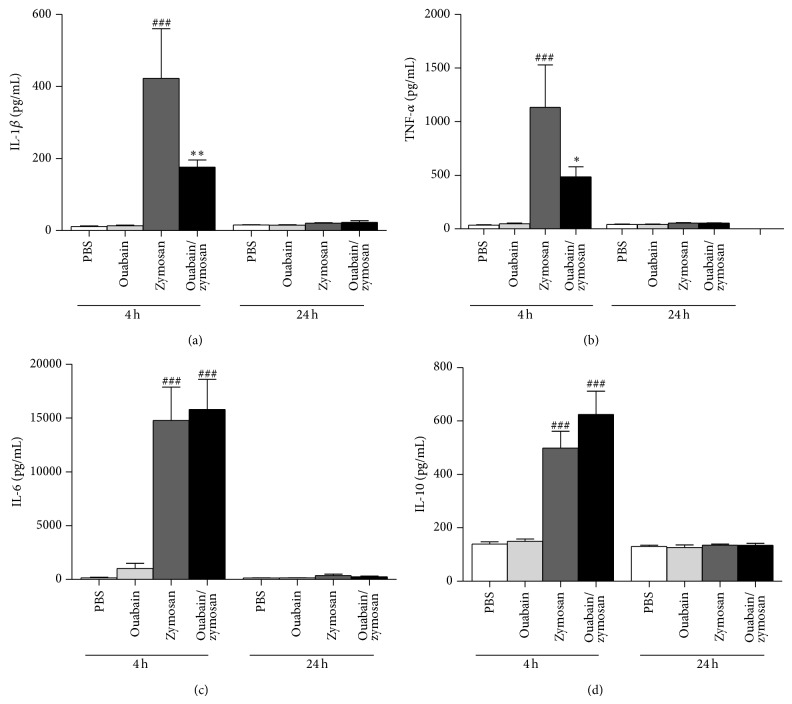
Effect of ouabain on proinflammatory and anti-inflammatory cytokines levels. Swiss mice (*n* = 8) were treated for three consecutive days with 0.56 mg/kg of ouabain or vehicle (PBS). After 4 and 24 h stimulation with zymosan, the peritoneal exudate was collected, centrifuged, and stored at −20°C until cytokine assay. Levels of IL-1*β* (a), TNF-*α* (b), IL-6 (c), and IL-10 (d) were measured in the exudate supernatant by ELISA. Results were expressed as mean ± SEM and analyzed by Graphpad Prism using ANOVA with Tukey's posttest, where all groups were compared. ^###^ *P* < 0.001 versus PBS group; ^∗^ *P* < 0.05 and ^∗∗^ *P* < 0.01 versus zymosan group.

Reduced neutrophil migration promoted by ouabain could be a consequence of the modulation of cytokines levels (TNF-*α*, IL-1*β*, IL-6, and IL-10) at 4 and 24 h after zymosan stimulation.  Figure 4 indicates that animals stimulated with zymosan showed increased levels of TNF-*α*, IL-1*β*, IL-6, and IL-10 (96%, 97%, 99%, and 72%, resp.) in the peritoneal cavity at 4 h, but these levels returned to baseline values at 24 h. Ouabain pretreatment for three consecutive days reduced significantly IL-1*β* (58%) and TNF-*α* (57%) levels (Figures 4(a) and 4(b)) at 4 h, but not at 24 h, when compared to the zymosan group. On the other hand, ouabain did not affect IL-6 and IL-10 levels when compared to zymosan group at both time points studied (Figures 4(c) and 4(d)).

### 3.5. Effect of Ouabain on the Number of Apoptotic and Necrotic Leukocytes in the Peritoneum

**Figure 5:**
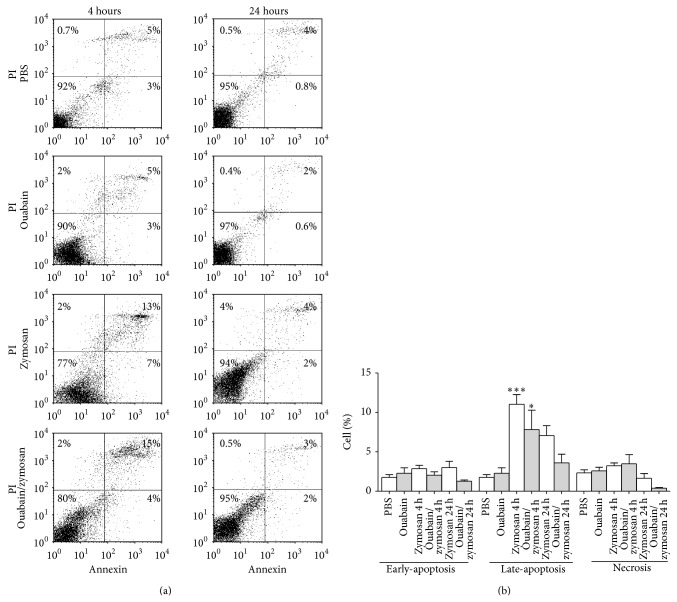
Effect of ouabain on peritoneal leukocytes viability. Viability of the leukocytes present in peritoneal lavage was assessed by flow cytometry. For that, cells obtained by peritoneal lavage were stained with Annexin V and propidium iodide in order to evaluate the percentage of cells undergoing apoptosis and necrosis, respectively. (a) Representative* dot plots* showing the percentage of cells undergoing initial apoptosis (Annexin V^+^), late-apoptosis (Annexin V^+^ and PI^+^), and necrosis (PI^+^). (b) Data were expressed as mean ± SEM and analyzed by Graphpad Prism using ANOVA with Tukey's posttest, where all groups were compared. Results were obtained from 6 animals per group. ^∗∗∗^ *P* < 0.001 and ^∗^ *P* < 0.05 versus PBS group.

To investigate a possible modulation of apoptosis by ouabain at 4 and 24 h, the percentage of apoptotic and necrotic leukocytes in the peritoneum was accessed by flow cytometry. Mice peritoneal cavity injected with PBS contained a low percentage of apoptotic and necrotic cells, and these numbers were increased by zymosan injection at 4 and 24 h compared with PBS group (Figure 5). Our results demonstrated that preinjection with ouabain does not interfere in the death of these cells at the times evaluated (Figure 5).

### 3.6. Effect of the Ouabain on Macrophage Viability and FITC-Dextran Endocytosis

**Figure 6:**
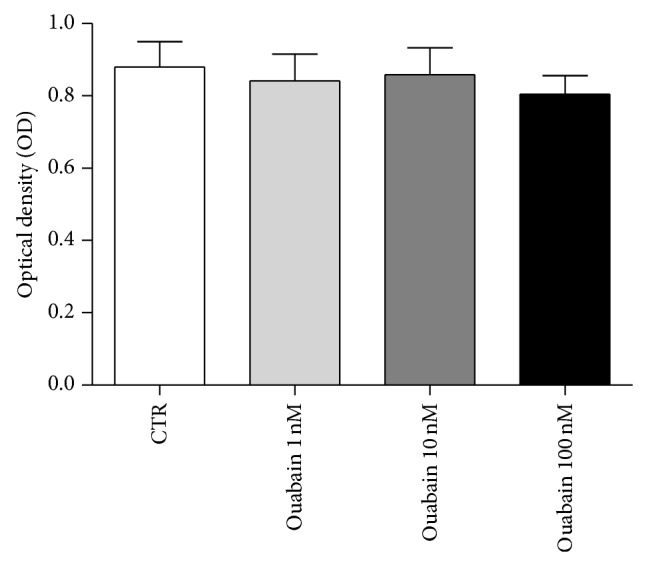
Effect of the ouabain on the viability of peritoneal macrophages. Analysis of macrophage viability was assessed by MTT reduction method. The graph represents the mean ± SEM of at least three independent experiments performed in duplicate.

**Figure 7:**
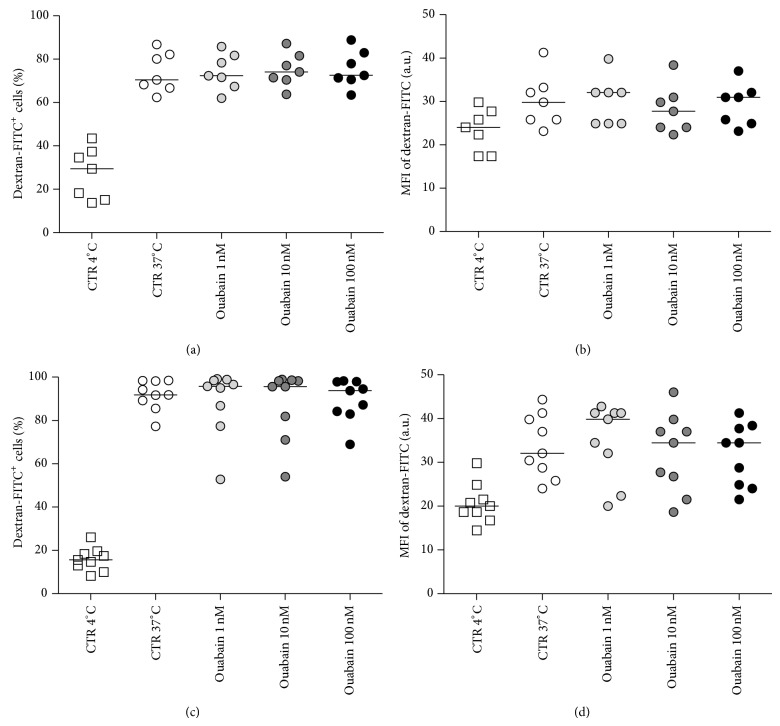
Effect of ouabain on* in vitro* dextran endocytosis by resident peritoneal leukocytes. ((a), (b)) Resident peritoneal cells were incubated with 1, 10, and 100 nM ouabain and 0.5 mg/mL of FITC-conjugated dextran for 1 hour at 37°C or 4°C (control endocytic activity), and fluorescence signals were analyzed by flow cytometry. Scatter plots indicate the percentage of dextran-FITC^+^ cells (a) and the median fluorescence intensity of dextran-FITC (b). ((c), (d)) Prior to the endocytic assay, resident peritoneal cells were cultured in the absence or presence of ouabain for 24 hours. After this culture period, these cells were then incubated for 1 hour with FITC-conjugated dextran at 37°C or 4°C, and fluorescence signals were analyzed by flow cytometry. Scatter plots indicate the percentage of dextran-FITC^+^ cells (c) and the median fluorescence intensity of dextran-FITC (d). Lines refer to the median of the results obtained from at least 7 animals per group.

Direct ouabain cytotoxicity for murine thioglycolate-elicited peritoneal cells was studied* in vitro*. Our results show that ouabain (1, 10, and 100 nM) did not interfere with macrophages viability (Figure 6). Additionally, we tested ouabain effect on FITC-dextran endocytosis by resident peritoneal cells. No significant difference was observed on the percentage of dextran-FITC^+^ cells or on the mean fluorescence intensity of endocytic cells, indicating that ouabain did not affect the number of endocytic cells or the amount of dextran particles endocytosed by them (Figure 7). Also, no significant difference was observed on FITC-dextran endocytosis by peritoneal cells from thioglycolate-elicited mice (data not shown).

### 3.7. Ouabain Inhibits Cell Migration

The diminished number of cells in the peritoneal cavity of zymosan injected mice pretreated with ouabain could be a result of an effect on the migratory capacity of cells from ouabain-treated animals. To investigate this possibility, migratory responses were assessed using a transwell cell migration assay. Our preliminary experiment shows a reduction on the number of migrating peritoneal cells of ouabain pretreated mice group when compared to control group (PBS-injected mice). As an* in vitro* attractant LPS (100 ng/mL) was used in the lower chamber of transwell plates. The number of migrating peritoneal cells from untreated mice towards culture medium was 19 × 10^3^ and towards culture medium plus LPS was 35 × 10^3^, whereas the number of migrating peritoneal cells from ouabain pretreated mice towards culture medium was 6 × 10^3^ and towards culture medium plus LPS was 11 × 10^3^.

### 3.8. Ouabain Modulates NF-*κ*B Transcription Factor

**Figure 8:**
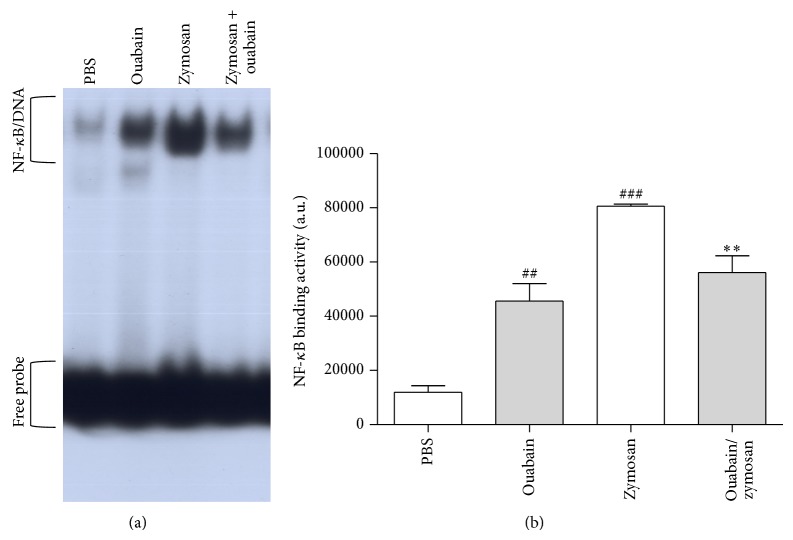
Ouabain reduced zymosan-induced NF-*κ*B binding activity. Electrophoretic mobility shift assay (EMSA) from peritoneal cells 4 h after injection with zymosan. Nuclear proteins (5 *μ*g) were extracted from peritoneal cells after 4 h injection with zymosan or PBS. (a) Protein/DNA complexes positions are indicated by arrows (NF-*κ*B) and the nonspecific band (NS) is also indicated. The respective densitometric analyses (arbitrary units (a.u.)) of the NF*κ*B band complex presented in panel (a) are shown in graphic (b). Results were expressed as mean ± SEM and analyzed by Graphpad Prism using ANOVA with Tukey's posttest, where all groups were compared. Results were obtained from 4 animals per group. ^##^ *P* < 0.01 and ^###^ *P* < 0.001 versus PBS group and ^∗∗^ *P* < 0.01 versus zymosan group.

Finally, we investigated whether ouabain could modulate NF-*κ*B activity in peritoneal cells of mice exposed or not to zymosan (Figure 8). To verify DNA-binding activity of NF-*κ*B, EMSA was performed using nuclear proteins isolated from peritoneal cell. Our results show that the binding activity of NF-*κ*B was increased (85%) in peritoneal cells from zymosan-treated mice at 4 h when compared to control group animals. Furthermore, ouabain significantly reduced (30%) zymosan-induced NF-*κ*B binding activity. Moreover, ouabain by itself also increased (73%) NF-*κ*B binging activity in peritoneal cells when compared to PBS group.

## 4. Discussion

In previous studies, our group demonstrated, using intraplantar injection of different phlogistic agents, an* in vivo* anti-inflammatory and analgesic potential of ouabain, which might be related to prostaglandin E2 as well as to opioid mechanisms [18]. It was also demonstrated that ouabain negatively modulated acute peritoneal inflammatory response induced by* Leishmania amazonensis* infection by decreasing TNF-*α* and IFN-*γ* levels [17]. In order to better understand the mechanisms involved in the anti-inflammatory effect of ouabain, a zymosan-induced peritonitis model was used in the present work. In accordance with what was previously reported, ouabain produced an anti-inflammatory effect which involves a reduction of IL-1*β* and TNF-*α* levels, reduced neutrophil migration, and decreased vascular permeability.

Intraperitoneal injection of zymosan increases vascular permeability, one of the primary signs of inflammation, within 30 minutes after the inflammatory stimulus due to the activation of resident macrophages and mast cells, which release prostaglandins, particularly histamine and leukotrienes [26, 28–30]. The reduction of the increase in vascular permeability promoted by ouabain appears to be related to mast cell degranulation and action PGE2, since ouabain treatment was capable of decreasing paw edema induced by compound 48/80 and PGE2 [18]. Furthermore, in accordance with our findings,* in vitro* studies demonstrated that ouabain inhibited histamine release from mast cells [35].

Zymosan injection in Swiss mice induces massive polymorphonuclear leukocytes (PMN) influx with maximal cell accumulation at the 4th hour of peritonitis [27, 28, 32]. In the present study, ouabain pretreatment for three consecutive days produced anti-inflammatory effects, decreasing the number of intraperitoneal PMNs at the 4th hour. These data are consistent with earlier studies by our group, where ouabain reduced the number of PMNs in models of peritonitis induced by Concanavalin A and* Leishmania amazonensis* [17, 18]. Our results show a reduction of the number of peritoneal cells of ouabain pretreated mice group, suggesting that inhibition of cell migration may be a key event for the ouabain-induced decrease in the number of peritoneal leukocytes observed by us. Furthermore, our preliminary* in vitro* study using a transwell model indicated that peritoneal cavity cells from ouabain treated mice migrate less than those of control animals. In addition, other studies have demonstrated that ouabain inhibits* in vitro* neutrophil migration, by interfering with IL-8 receptor recycling [36]. Besides that, oleandrin, another cardiac glycoside, potentially inhibited IL-8-mediated biological responses in diverse cell types [37]. Furthermore, in studies with lung tumor cells, cardiotonic glycosides, ouabain, and odoroside A inhibited ICAM-1 adhesion molecule expression [38].

Inflammatory signaling in response to zymosan, which can be also recognized by dectin and complement receptors [39, 40], requires TLR2/TLR6 heterodimerization and subsequent recruitment to the zymosan phagosome [41]. This action is essential for nuclear factor NF-*κ*B activation. NF-*κ*B is a pivotal regulator in the expression of many proinflammatory cytokines such as tumor necrosis factor- (TNF-) *α*, interleukin- (IL-) 1*β*, IL-6, IL-10 chemokines, and NO [42, 43] leading the leukocytes infiltrating zymosan-inflamed peritoneum such as neutrophils and macrophages. We investigated whether the inhibitory effect of ouabain preinjection was related to modulation of cytokine levels and NF-*κ*B activation. In this model, our results demonstrated that treatment with ouabain inhibited TNF-*α* and IL-1*β* production induced by zymosan, but not IL-6 and IL-10 levels. However, other groups described that ouabain suppressed the production of the proinflammatory cytokines IL-6 and TNF-*α* stimulated by LPS both* in vitro* and* in vivo* [21]. The difference between our results and those cited above may be due to a different nature of the inflammatory agent (fungal), the source of investigated samples (peritoneal exudates versus serum), and ouabain concentrations. In addition, it has been reported that cardiac glycoside drugs inhibit TNF-*α*/NF-*κ*B signaling pathway, which is a central common regulator for inflammatory process [44]. In the present study, when ouabain was associated with zymosan it significantly decreased the NF-*κ*B activation observed with zymosan alone. However, similar to a study using dendritic cells [16] ouabain by itself was capable of increasing NF-*κ*B activation. Therefore, the decrease in TNF-*α* and IL-1*β* levels observed by us correlates with an inhibitory effect of ouabain on NF-*κ*B binding activity. Furthermore, other studies with LPS-stimulated PBMC cells indicated that digitalis including ouabain, proscillaridin A, digoxin, digitoxin, and lanatoside C reduced TNF-*α*, IL-1*β*, and IL-6 production by inhibiting NF-*κ*B signaling pathway [44]. Moreover, ouabain was able to inhibit IL-1*β* release in primary astrocytes stimulated with LPS [45]. Another cell signaling pathway involved in the production of proinflammatory cytokines, the p38 MAPK pathway, is also regulated by ouabain [13, 46].

Zymosan peritonitis represents a model of acute resolving inflammation [25, 26, 28, 31, 42]. A fundamental paradigm of inflammatory research has postulated that neutrophil apoptosis and its subsequent clearance by macrophages are the major mechanisms promoting resolution of inflammation [47, 48]. Ouabain treatment did not interfere with peritoneal cavity leukocyte apoptosis. These data may be associated with ouabain's ability to reduce TNF-*α* production, which is important for apoptosis induction of this cell [36]. Furthermore, it is known that digitalis inhibits TRADD receptor protein coupling, which leads to TNFR1 inhibition [44]. The process of clearance involves endocytosis. In the present work it was possible to observe that ouabain did not alter* in vitro* endocytosis of dextran particles by peritoneal cells. Thus, we suggest that the anti-inflammatory effects observed by us are not related to events involved in resolution; nevertheless more detailed studies should be performed to confirm this hypothesis.

## 5. Conclusion

Ouabain modulated acute inflammatory responses induced by zymosan, reducing IL-1*β* and TNF-*α* levels and the number of peritoneal cells, possibly due to a decrease in cell migration. Moreover, ouabain inhibited NF-*κ*B activation, which is related to the production of TNF-*α* and IL-1, cytokines important for leucocyte extravasation. Taken together, our results provide new evidences for the mechanisms related to the anti-inflammatory effects of ouabain* in vivo*.
